# The Relationship between Teacher Autonomy and Mental Health in Primary and Secondary School Teachers: The Chain-Mediating Role of Teaching Efficacy and Job Satisfaction

**DOI:** 10.3390/ijerph192215021

**Published:** 2022-11-15

**Authors:** Yujue Peng, Huimin Wu, Cheng Guo

**Affiliations:** Research Center of Mental Health Education, Faculty of Psychology, Southwest University, Chongqing 400715, China

**Keywords:** teacher autonomy, teaching efficacy, job satisfaction, mental health

## Abstract

Teachers in primary and secondary schools are frequently under pressure. Therefore, it is critical to understand the factors that affect their mental health. Autonomy was associated with mental health in the past. However, the mediating mechanism behind this relationship has received little attention. In this study, a chain mediation model was built to determine whether teaching efficacy and work satisfaction mediated the relationship between teacher autonomy and mental health. Our study enlisted the participation of 810 Chinese primary and secondary school teachers aged from 21 to 57 years old. They completed self-reporting measures of teacher autonomy, mental health, teaching efficacy, and job satisfaction. The results show that (1) teacher autonomy, teaching efficacy, job satisfaction, and mental health have strong positive relationships, (2) teaching efficacy and job satisfaction significantly mediate the relationship between autonomy and mental health, and (3) both teaching efficacy and job satisfaction play a chain-mediating role. The chain-mediating effect of teaching efficacy and job satisfaction plays an important role in promoting teachers’ mental health. Teachers with a high level of autonomy tend to have high teaching efficacy, high job satisfaction, and improved mental health.

## 1. Introduction

Teaching is a special profession because it is the responsibility of teachers to teach and educate people. It is also a profession that carries high social expectations and family pressures because of its special nature. With the advancement of many educational reforms, the workload of the teaching profession has become heavier, and the pressure faced by teachers has increased, which has led to many mental health problems that affect teachers’ work and lives. Although more attention is being paid to mental health education in primary and secondary schools, more attention is often paid to the mental health of students and less to the mental health of teachers. High levels of teaching stress can lead to a continued deterioration in teachers’ mental health, which may negatively impact student health and educational achievement [1,2]. Previous studies have shown that teachers’ mental health issues have negative effects on their teaching efficacy [3]. In addition, teachers’ mental health is related to students’ futures. For instance, a study showed that teachers’ stress can affect students’ mental health through negative emotions and perceptions [4]. Mental health status is very important for teachers and critical to educational excellence [5]. Therefore, it is essential to pay special attention to teachers’ mental health.

Recognizing this importance, and drawing on data from a questionnaire, the present study moves toward contributing to the improving of primary and secondary school teachers’ mental health, taking primary and secondary school teachers as an example. Although this study focuses on primary and secondary school teachers, it may have wider implications in other similar contexts. In light of the foregoing, this study seeks to address the research question of how to improve primary and secondary school teachers’ mental health.

### 1.1. Teacher Autonomy and Mental Health

In modern psychology, many terms can be used to describe mental health, such as happiness, life satisfaction, and well-being [6]. Life satisfaction is a mental health index defined as a person’s attitude toward his life in general or a particular aspect of it, such as family, occupation, or education [7]. A previous study showed that autonomy has a significant influence on life satisfaction [8], suggesting that autonomy might influence mental health.

The job demand-control model suggests that job-related psychological stress can be explained by the interaction of two work characteristics: demand, which includes workload, stress, and other factors, and control, which includes autonomy and decision making [9]. Autonomy is an individual’s ability to act as he or she wishes with less reliance on others and the environment [10]. Therefore, autonomy can also be understood as the ability to decide how to live one’s life and plays a fundamental role in an individual’s mental health [11,12]. People experience more negative feelings when faced with a job that is low in autonomy but high in emotional labor [13,14]. Teacher autonomy means that teachers can be given the freedom to provide the best learning solution for their students [15]. When teachers are given great autonomy, they tend to feel understood, respected, and encouraged in their advocacy [16]. They experience less conflict [17] and more happiness, satisfaction, interest, pride, gratitude, and love [18], which contribute to their mental health. Therefore, we speculate that teacher autonomy is significantly correlated with teachers’ mental health.

### 1.2. Mediating Role of Teaching Efficacy

There are two types of expectations playing a large role in influencing people’s choice of activity or their efforts to achieve a goal: outcome expectations, wherein people estimate the outcome that a behavior will bring, and efficacy expectations, wherein people believe they will be able to successfully accomplish the behavior needed to carry out tasks [19]. Extending Bandura’s concept of efficacy expectations to education, teaching efficacy is defined as teachers’ judgment of their ability to bring the expected teaching effect to students [20]. Teaching efficacy is usually categorized into two types: personal and general teaching efficacy. Personal teaching efficacy is more closely linked to instructors’ beliefs in their own ability to teach, whereas general teaching efficacy is the teachers’ expectations of the impact their teaching activities will have on student learning [21,22,23]. Prior research has found that teachers with a high sense of teaching efficacy are more resilient in their teaching and are likely to try harder to help students reach their potential [24]. On the other hand, teachers with low teaching efficacy tend to adopt avoidance strategies in the face of frustration and show higher levels of anxiety and depression due to student misbehavior and poor student discipline [25]. Therefore, beliefs of teaching efficacy are the most central and pervasive among the mechanisms of teaching, and teachers are motivated to act because they want to achieve their desired purposes through their actions. This implies that teaching efficacy may be closely related to teachers’ mental health. Thus, based on this evidence, we can infer that teacher autonomy can influence mental health through teaching efficacy.

### 1.3. Mediating Role of Job Satisfaction 

Job satisfaction can be defined as “a pleasurable or positive emotional state resulting from the appraisal of one’s job or job experiences” [26]. Among the factors influencing job satisfaction, studies have examined the effect of job autonomy on job satisfaction, suggesting that workers who enjoy a higher degree of autonomy at work have higher job satisfaction [27]. Other studies have also found that job autonomy is positively correlated with job satisfaction [27], and if staff have high autonomy, then they also have greater job satisfaction and mental happiness [28]. For example, in a survey of medical workers in the context of COVID-19, it was found that the higher their job satisfaction, the more they wanted to perform the job, and the higher their physical and mental health [29]. This may also be the case for teachers. This means that teachers will be more satisfied with their jobs if they find more autonomy in working. Therefore, based on empirical evidence, we infer that the impact of teacher autonomy on mental health is mediated by job satisfaction.

### 1.4. Chain-Mediating Role of Teaching Efficacy and Job Satisfaction

**Figure 1:**
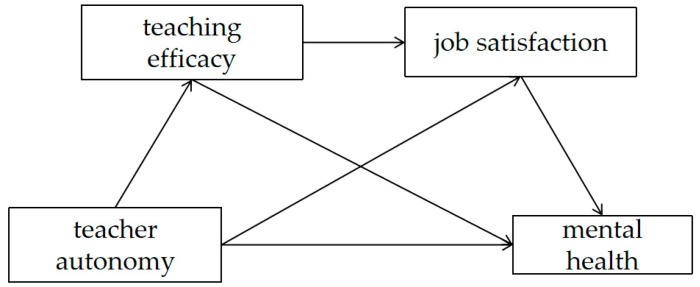
Hypothesized model.

As hypothesized, teaching efficacy and job satisfaction mediate the relationship between teacher autonomy and mental health. However, the relationship between teaching efficacy and job satisfaction and its mediating effects on teacher autonomy and mental health remain unknown. It was concluded in previous studies that teachers’ self-efficacy and job satisfaction are positively correlated [30,31,32,33]. The relationship between teacher autonomy and mental health may be influenced first by the factor of teaching efficacy and then by job satisfaction. The authors of [34] suggested that teacher efficacy can be considered significant if teachers’ job satisfaction increases. It can be said that teachers’ consideration of themselves as persons with teaching efficacy may positively influence their job satisfaction. Therefore, we infer that the impact of teacher autonomy on mental health is mediated by teaching efficacy and job satisfaction. Specifically, we inferred that both teaching efficacy and job satisfaction have a chain-mediating effect on the relationship between teacher autonomy and mental health (see Figure 1).

## 2. Method

### 2.1. Participants

The participants in this study were 810 teachers (81.23% were females and 18.14% were males) aged from 21 to 57 years old who worked in different primary and secondary schools in China. The questionnaires were distributed through the online platform “Questionnaire Star”. The online questionnaire was circulated in training programs to ensure the authenticity of the respondents’ identities. 

### 2.2. Measures 

The online questionnaire consisted of four scales measuring teacher autonomy, mental health, teaching efficacy, and job satisfaction. The descriptions of the four scales follow.

#### 2.2.1. Teacher Autonomy 

Teacher autonomy was measured using the Teacher Autonomy in Primary and Middle Schools Scale [18]. Each item was rated on a 5-point Likert scale ranging from 1 (not at all true of me) to 5 (very true of me), with a higher score indicating a higher level of teacher autonomy. The scale includes 20 items measuring autonomous willingness, choice, behavior, and experience. The scale showed good reliability (Cronbach’s α = 0.964).

#### 2.2.2. Mental Health

Mental health was measured using the Mental Health Test (Ⅱ) [30], which is referred to as the CAS. The scale includes 40 items that measure a person’s self-control, self-maturity, suspiciousness and delusion, guilt, and impulsive tension. The scale applies a 3-point scale ranging from 2 (yes) to 0 (no), where “1” means not sure. The results can be converted into 1–10 standard points, with higher scores indicating a more serious problem. In the current study, this scale showed good reliability (Cronbach’s α = 0.747). 

#### 2.2.3. Teaching Efficacy

Teacher self-efficacy was measured by a 27-item teacher’s sense of teaching efficacy scale [23]. The scale had two dimensions. The dimensions were general teaching efficacy and personal teaching efficacy. Responses were given on a 6-point scale from “do not agree at all” (1) to “absolutely agree” (6). Examples of items include “I can solve problems that arise in student learning” (personal teaching efficacy) and “Generally speaking, what a student becomes is predetermined” (general teaching efficacy). In the current study, the scale showed good reliability (Cronbach’s α = 0.915).

#### 2.2.4. Job Satisfaction

Job satisfaction was measured using a four-item scale used in previous research [35]. The items were as follows: “I am happy to come to work”, “I want to continue for a long time in my current workplace”, “My current job is rewarding”, and “I enjoy being in my current job position”. Responses were given on a 6-point scale ranging from 1 (strongly disagree) to 6 (strongly agree). In the current study, the scale showed good reliability (Cronbach’s α = 0.927).

### 2.3. Data Analysis

SPSS 25.0 and Mplus 7.0 were used to analyze the data. First, Pearson’s correlations were found to determine the associations between teacher autonomy, teaching efficacy, job satisfaction, and mental health. Second, a structural equation model was constructed to test the mediating effect of teaching efficacy and job satisfaction in the relationship between teacher autonomy and mental health. Latent moderate structural equations were used to test the moderating effects of job satisfaction on the relationship between professional identity and burnout. The fit of the model was evaluated using the following indices: the root mean square error of approximation (RMSEA), standardized root means square residual (SRMR), comparative fit index (CFI), and the Tucker–Lewis index (TLI). The following criteria were used to indicate the goodness of fit: TLI ≥ 0.90, CFI ≥ 0.90, RMSEA ≤ 0.10, and SRMR ≤ 0.10.

## 3. Results

### 3.1. Common Method Bias

The results of Harman’s single factor test were used to test the common method bias effect. The results show that the first principal component explained 21.43% of the total variance, which was less than 40% [36], indicating that common method bias was not a serious problem in this study.

### 3.2. Descriptive Statistics and Correlation

Correlation analyses showed that teacher autonomy was significantly positively correlated with their scores for mental health, teacher efficacy, and job satisfaction (see Table 1).

### 3.3. Structural Model

The statistical significance of these hypothesized indirect effects on relationship stability was tested using the bias-corrected bootstrap sampling procedure available in Mplus7. First, teacher autonomy significantly predicted mental health (β = 0.281, t = 8.333, *p* < 0.001). The chain-mediating model demonstrated a good model fit, with χ2/df = 5.108, RMSEA = 0.071, CFI = 0.957, TLI = 0.947, and SRMR = 0.048. The total indirect effects of the three variables on mental health are shown in Table 2. The indirect effect of teacher autonomy on mental health was significant: (1) The indirect effect of teacher autonomy on mental health via teaching efficacy was significant (indirect effect = 1.502, 95% CI [0.068, 0.274]), (2) the indirect effect of teacher autonomy on mental health via job satisfaction was also significant (indirect effect = 0.488, 95% CI [0.007, 0.104]), and (3) we found that the indirect effect of teacher autonomy via teaching efficacy and job satisfaction on mental health was positive and significant (*p* < 0.05), while the 95% confidence interval (indirect effect = 0.319, 95% CI [0.233, 0.405]) excluded zero. Additionally, the 95% bias-corrected confidence interval for the direct effect of teacher autonomy on mental health was zero (direct effect = −0.002, 95% CI [−0.116, 0.112]).

**Figure 2:**
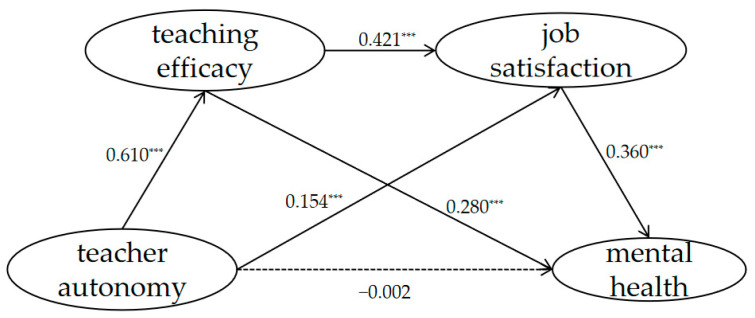
Serial mediation model testing teaching efficacy and job satisfaction as mediators between teacher autonomy and mental health; *** p < 0.001.

Importantly, teaching efficacy and job satisfaction mediated the link between teacher autonomy and mental health (see Table 2). Overall, the hypotheses were partially supported (see Figure 2).

## 4. Discussion

In the present study, we focused on the mental health of primary and secondary school teachers. Our study explored the relationship between teacher autonomy and the mental health of primary and secondary school teachers, as well as the mediating effect of teacher autonomy and job satisfaction. A study used linear regression to find that teacher autonomy positively predicted mental health, which also largely solidified the relationship between teacher autonomy and mental health [18]. In line with our expectations, the results showed that teacher autonomy indirectly influenced mental health via three pathways—teaching efficacy, job satisfaction, and the chain-mediating effect of teaching efficacy and job satisfaction—which contributes to a deeper understanding of the mechanism between teacher autonomy and mental health.

Teachers’ autonomy can positively predict mental health. Autonomy refers to the feeling of choosing one experience when one’s actions are consistent with his or her self-approved values and interests [37]. This means that teachers can choose what they want to achieve in their teaching, which would be worthy for them. Combined with the job demand-control model, a study confirmed that job demand is related to workers’ mental health, and the interaction of job demand and control affects workers’ mental health, such as depression and anxiety [38]. Therefore, if teachers have a high level of autonomy, then this would be helpful for their mental health.

Furthermore, this study showed that teaching efficacy can influence the quality of teachers and the effectiveness of teaching. Teachers with high teaching efficacy are more resilient in their teaching and are likely to try harder to help all students reach their potential [20]. This study also found that teaching efficacy played a partial mediating role in the relationship between teacher autonomy and mental health. This is consistent with the following findings. First, individuals seek a sense of control over their environment to enhance their well-being [39]. For teachers, job autonomy can give them a sense of control and provide them with the opportunity to realize their personal values [40], which in turn improves their teaching efficacy. Second, another study suggested that teachers with low teaching efficacy would have a lower sense of competence [41]. In conjunction with the self-determination theory, competence is one of the basic needs, and failure to meet the need for competence tends to reduce an individual’s mental health. Therefore, we found that teacher autonomy can affect mental health through teaching efficacy.

Our results not only verified the mediating effect of teaching efficacy, underlying the relationship between teacher autonomy and mental health, but also confirmed the mediating effect of job satisfaction. This is in line with the results of prior studies [33,42], which showed that teacher autonomy could positively predict teachers’ job satisfaction. This result is also consistent with the findings of a study that showed that job satisfaction could significantly predict well-being [43], and this means that job satisfaction can predict mental health. Thus, job satisfaction may be a mediator between teacher autonomy and mental health. Furthermore, a significant path of teacher autonomy → teaching efficacy → job satisfaction → mental health was found. This model illustrates that teaching efficacy acts as a mediator between teacher autonomy and mental health, while job satisfaction mediates the relationship between teacher autonomy and mental health. Previous studies have supported the finding that teachers’ sense of self-efficacy positively predicts their job satisfaction [32]. In other words, the chain-mediating effect of teaching efficacy and job satisfaction suggests that teachers with high-level autonomy would report that they have more efficacy in teaching, which may give teachers a higher level of job satisfaction and contribute to good mental health.

This study has several strengths and limitations that offer directions for future research. One strength of our study is that we tested a large sample size of primary and secondary school teachers and tested their mental health and teacher autonomy at the present time. Another strength is that this study identified the mediators that explain how teacher autonomy influences mental health. Future studies could consider teacher autonomy and provide a new method to improve teachers’ mental health. Despite its strengths, this study has several limitations. First, it had a cross-sectional design, which does not allow establishing the causality of mediation. Longitudinal studies can be conducted using the factors of this study to determine how and why the factors change over time. Second, we collected fewer data on men than women in the sociodemographic variables, which limited the possibility to explore how the mediation model worked with different groups of people. Despite these limitations, we believe that the present study contributes to filling this gap in our knowledge of teacher autonomy and mental health.

## 5. Conclusions

This study explored the relationship between teacher autonomy and the mental health of primary and secondary school teachers. It also concluded that mental health in primary and secondary schools is closely related to teacher autonomy, teaching efficacy, and job satisfaction. The study extends the findings of previous studies that (1) teaching efficacy mediates the relationship between teacher autonomy and mental health, (2) job satisfaction mediates the relationship between teacher autonomy and mental health, and (3) both teaching efficacy and job satisfaction have a chain-mediating effect on the relationship between teacher autonomy and mental health.

This study highlights the importance of focusing on improving teachers’ mental health by improving teacher autonomy, teaching efficacy, and job satisfaction. This study may be able to inform future practices because little is known about the relationship of teaching efficacy and job satisfaction with autonomy and mental health, as well as the interactions between them. Therefore, the results of this study can be used to guide policies and strategies to improve teachers’ mental health. For example, educational administrators should attach great importance to teachers’ mental health and firmly establish the concept of teachers not only being workers but also educational leaders. In addition, teachers can be given full support and autonomy so that they can become lovers of their own work and devote themselves to teaching and educating people in a relaxed, free, and enthusiastic state.

## Figures and Tables

**Table 1:** Descriptive statistics and correlations among study variables.

Variables	M (SD)	1	2	3	4
1.Teacher autonomy	4.049 (0.599)	-			
2. Mental health	5.500 (0.526)	0.281 **	-		
3. Teacher efficacy	4.448 (0.664)	0.482 **	0.418 **	-	
4. Job satisfaction	4.961 (0.984)	0.394 **	0.421 **	0.495 **	-

Note. ** p < 0.01.

**Table 2:** Estimated and standard errors for the serial mediation model.

Pathway	Est	SE	Bias-Corrected 95% CI		
			Lower	Upper	*p*
TA→TE→JS→MH	0.093	0.019	0.056	0.129	0.000
TA→TE→MH	0.171	0.068	0.068	0.274	0.001
TA→JS→MH	0.055	0.024	0.007	0.104	0.023
Indirect	0.319	0.044	0.233	0.405	0.000
Direct	−0.002	0.058	−0.116	0.112	0.976

Note. TA = teacher autonomy, TE = teaching efficacy, MH = mental health, JS = job satisfaction, 95% CI = 95% confidence intervals of bias-corrected bootstrapping. Mental health used the sten score, and all coefficients were standardized in Mplus 7.0.

## Data Availability

Data are available from the corresponding author upon reasonable request.
